# Transportation Mode Detection Using Learning Methods and Self-Contained Sensors: Review

**DOI:** 10.3390/s24227369

**Published:** 2024-11-19

**Authors:** Ilhem Gharbi, Fadoua Taia-Alaoui, Hassen Fourati, Nicolas Vuillerme, Zebo Zhou

**Affiliations:** 1 GIPSA-Lab, Univ. Grenoble Alpes, CNRS, Inria, Grenoble INP, 38000 Grenoble, France; fadoua.taia-alaoui@univ-grenoble-alpes.fr (F.T.-A.); hassen.fourati@gipsa-lab.fr (H.F.); 2 AGEIS, Univ. Grenoble Alpes, 38000 Grenoble, France; nicolas.vuillerme@univ-grenoble-alpes.fr; 3 GRICAD, CNRS, Univ. Grenoble Alpes, 38000 Grenoble, France; 4 School of Aeronautics and Astronautics, University of Electronic Science and Technology of China, Chengdu 611731, China; klinsmann.zhou@gmail.com

**Keywords:** transportation mode detection, machine learning, classification, inertial sensors, smartphones

## Abstract

Due to increasing traffic congestion, travel modeling has gained importance in the development of transportion mode detection (TMD) strategies over the past decade. Nowadays, recent smartphones, equipped with integrated inertial measurement units (IMUs) and embedded algorithms, can play a crucial role in such development. In particular, obtaining much more information on the transportation modes used by users through smartphones is very challenging due to the variety of the data (accelerometers, magnetometers, gyroscopes, proximity sensors, etc.), the standardization issue of datasets and the pertinence of learning methods for that purpose. Reviewing the latest progress on TMD systems is important to inform readers about recent datasets used in detection, best practices for classification issues and the remaining challenges that still impact the detection performances. Existing TMD review papers until now offer overviews of applications and algorithms without tackling the specific issues faced with real-world data collection and classification. Compared to these works, the proposed review provides some novelties such as an in-depth analysis of the current state-of-the-art techniques in TMD systems, relying on recent references and focusing particularly on the major existing problems, and an evaluation of existing methodologies for detecting travel modes using smartphone IMUs (including dataset structures, sensor data types, feature extraction, etc.). This review paper can help researchers to focus their efforts on the main problems and challenges identified.

## 1. Introduction

With the constant evolution of smartphones, the tracking of human activities has notably expanded [1,2], facilitating the development of intelligent transportation systems and smart city applications [3,4,5,6]. There are many studies on transportation mode detection (TMD) systems, of which we can cite the project InnaMoRuhr [7], funded by the North Rhine-Westphalia (NRW) ministry of transport, that was conducted from September 2022 to January 2023. This project focused on the improvement of the durability of mobility in Ruhr area by developing mobile applications that enable users mobility monitoring. Thanks to the prevalence of smartphones and their embedded sensors [8,9,10,11], along with communication and computing capabilities, TMD applications can collect, transmit and analyze data in real time [12], providing users with practical and effective information [13,14]. Reliable recognition of transportation modes leads to a variety of practical applications, such as the implementation of more informative studies on modes of transportation, optimizing urban organization and traffic flow management [15], encouraging public transport usage [16,17], reducing CO_2_ emissions [18], optimizing localization algorithms and estimating travel times for different types of vehicles with a better accuracy [12].

Some review papers exist in the literature and address TMD systems and related learning approaches [19,20,21]. They primarily aim to provide an overview of the used methods without delving into the details and highlighting the difficulties encountered from data collection to classification. We consider this aspect to be the most relevant when evaluating the performance of a TMD system. In this light, the aims of the proposed review are to provide an in-depth state-of-the-art review of modes detection using smartphone sensors based on recent references, to assess existing data processing methods and to identify various factors influencing the accuracy of TMD systems, such as the heterogeneous nature of datasets, sensor data types, the process of feature extraction and optimization, the type of IMUs (and related sensor quality) used to collect data and the challenges encountered with real-world data collection. This will give researchers a thorough understanding of the complexities and considerations surrounding TMD systems.

This paper’s outline is the following: Section 2 gives a comprehensive review of the state-of-the-art in TMD systems, handling key aspects such as data collection methods, challenges with real-world data, optimal window lengths for data segmentation, feature extraction techniques and optimization and classifiers used in TMD systems. We also analyze the evaluation metrics for performance characterization. Section 3 analyzes the various types and locations of sensors specially used in smartphones for TMD data collection. Section 4 provides an overview of existing Android applications for TMD data collection, evaluating their design and capabilities. Section 5 discusses the possibility of establishing a standardization framework. Finally, Section 6 provides a global conclusion about the findings of this survey.

## 2. State-of-the-Art for TMD Systems

**Figure 1:**
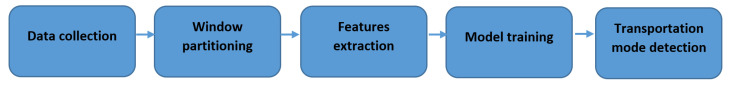
Processing pipeline for predicting the transportation modes.

Figure 1 shows the steps for predicting the transportation modes using the smartphone sensors. Data are first divided into segments with a sliding window. The data in each segment are used to compute a vector of features. These feature vectors are processed by a classifier used to predict the transportation modes.

In the following sections, we detail each step of the TMD process, from data collection to the classification of transportation modes. We analyze methodologies for data pre-processing, feature extraction as well as the application of machine learning algorithms for classification. Special attention will be given to challenges encountered with real data and some techniques proposed to address these challenges effectively.

### 2.1. TMD Data Collection

There are two methods of TMD data collection: either through IMU or through sensors integrated into the smartphone. The main smartphone sensors used to detect transportation modes are GPS, accelerometers, gyroscopes, magnetometers and barometers [22,23]. The following subsections describe the use of sensors in identifying different modes of transportation and illustrate existing public datasets.

#### 2.1.1. Main Sensors in TMD Systems

A GPS provides location data, real-time positioning, timing and velocity information [24]. Acceleration is calculated from a 3-axis accelerometer. It offers the possibility to choose the sampling frequency, enabling the user to find the optimal sampling rate through experiment [25]. The role of a gyroscope is to determine the rotation rate of the device based on the roll, pitch and yaw movements of the smartphone. The barometer measures the atmospheric pressure [23]. The value of pressure can be used to assess variations in pressure over time, such as those induced by pressure changes, elevation changes produced by tunnels, metros or airplanes [26]. A magnetometer gives a device’s orientation compared to the magnetic north of the Earth but it requires around twice as much battery consumption as a gyroscope [27] and detects ambient noise [28]. It is used in conjunction with other sensors, such as accelerometers to identify transport modes [29,30]. Combined with significantly more sensors, prediction results can be more accurate.

#### 2.1.2. Existing TMD Datasets

We reviewed existing datasets from the literature since 2018, as summarized in Table 1. Some datasets are private [31,32,33], but our focus is only on publicly accessible ones. Moreover, there are other older databases, but we focused on those published after 2018. Missing data are denoted “-”. The following notations were used for convenience:

- Sensors: L: Light, S: Sound, A: Accelerometer, G: Gyroscope, M: Magnetometer, B: Barometer, LA: Linear accelerometer, O: Orientation.
- Device: IMU: Inertial measurement unit, Mob: Mobile phone.
- Transportation modes: S: Still, W: Walk, R: Run, Sr: Stairs, E: Elevators, Bi: Bike, MC: Motorcycle, B: Bus, C: Car, T: Train, Tr: Tram, HSR: High speed rail, Sub: Subway, M: Metro, KS: Kick-Scooter, R: Run.

As for publicly available datasets, ordered from recent to old, the main datasets are the TMD-CAPTIMOVE [31,34], collected by 34 participants with a total duration of 48 h of data; the dataset [31], collected by 18 participants with a total duration of 140 h; the US-TMD dataset [35], collected by 13 participants with a total duration of 31 h and the SHL dataset [27], collected by three participants with a total duration of 703 h. We can see that the dataset TMD-CAPTIMOVE [34] is optimized in terms of balance between the number of participants and the total duration in addition to the introduction of electric and kick scooters as novel transportation modes which are lacking in the other datasets. This optimization not only improves the dataset’s applicability but also raises important considerations about the time scale of data collection. Broadly speaking, the time scale designates the time span of the data collection campaign. In fact, the more time separation there is between different experiments, the more the data are likely to cover different weather and traffic conditions, and therefore to provide sufficiently representative data samples. Out of the four datasets that were considered in this review [27,31,34,35], two studies provided this information [27,34]. One collected data during 3 months [34] and one during 7 months [27]. This information is missing in the two other studies [31,35], while the provided data show a significant heterogeneity with more or less long data collection periods. Moreover, the time scale can be approached through the total duration of the TMD dataset, which is the summed time length of all recorded signals, regardless of the data collection campaign time span. A TMD dataset with high total duration collected in a short time (e.g., a few consecutive days) is still likely to have less within-variance than data collected over a larger time scale due to redundant external conditions such as weather and traffic. On this aspect, the total time duration was indicated in all of the considered studies. It ranged from 31 h to 703 h. Globally, all the reviewed datasets have a total duration above 30 h. On the other hand, the minimum time duration (column 12 of Table 1: Minimum duration) dedicated to a given transportation mode should also be considered to provide insight about the balance or imbalance of the classes in terms of time distribution. But more importantly, it indicates whether there are enough train samples for a specific transportation mode. Indeed, the majority of TMD datasets are imbalanced [31,35], which is due to several factors. One reason for this imbalance is that, when indoor activities are included, they generally last less than outdoor activities. Therefore, it is normal to have fewer train samples for elevators and stairs, for example [34].

### 2.2. Challenges with Real-World Data Collection

The growing dependence on TMD systems for urban mobility solutions and smart city planning leads us to investigate the issues that may arise in the process of gathering data from sensors on real-world conditions. The following subsections will explain these challenges and propose some solutions.

#### 2.2.1. Variable Sampling Frequency

Multiple devices allows for choosing the preferred sampling frequencies when gathering sensor data. Due to activities taking place on smartphones that are not related to data collection, sampling frequencies are not constant over time, even once they have been set. The data collection application can be affected and its sampling frequency changed, for example, if another application consumes all the computing resources of the smartphone in the foreground, or if another application collects the same sensor data for TMD systems. Therefore, to address irregular sampling frequencies, the data will be subjected to down-sampling or up-sampling to a predetermined sampling frequency that the sensors can achieve, followed by a linear interpolation.

#### 2.2.2. Data Privacy Issues

Many data privacy issues arise when we handle data that track peoples’ routine mobility. The main problem is monitoring the precise location of users in order to detect their positions (for example, using GPS traces). This information concerns the privacy of users and contains private information. That is why, researchers have been focused on using accelerometers, gyroscopes, magnetometers and barometers that are able to detect transportation modes without violating privacy [36,37,38].

#### 2.2.3. Variable Smartphone Sensors

Based on the smartphone reference and performance, the sensor sets vary. For instance, some sensors in less expensive devices may record signals with less accuracy and quality. Additionally, in such type, smartphones may not have a barometer. In order to achieve good predictions, it is essential that the training dataset includes a variety of sensors with different qualities.

#### 2.2.4. Variable Circumstances in Data Collection

The signals that the sensors produce are notably affected by the circumstances in which the data are collected. For example, when driving on a highway at a constant speed, the car generates significantly different sensor data, suggesting that the user is stationary. Similarly, when driving on a dirt road at a constant speed, the road’s topography has a significant impact on the sensor signal output. As a solution, the signal can be filtered. However, this filtering may remove specific data artifacts that help differentiate a car from a bus. To address this problem, we can use an heterogeneous and diverse train dataset so that the model will perform better in a real-world scenario.

#### 2.2.5. Variable Smartphone Sensors Orientation

Users may keep their smartphones in different orientations when gathering sensor data, which leads to different data for each orientation. In addition, the orientation of the motion detection chips varies from one smartphone model to another. For instance, Apple devices have the *z*-axis sensors oriented in the opposite direction compared to the majority of Android devices. The magnitude metric is used as a solution [39] to extract orientation-independent features from sensor readings.

#### 2.2.6. TMD Data Quality

There are many types of errors encountered when recording data, such as outliers (also known as anomalies) [40], spikes [41], missing data [42], bias [42], drift [42] and noise [43]. Therefore, data quality issues need to be handled through data cleaning and pre-processing [44] such as imputation to fill in missing values, over-sampling for imbalanced data, denoising, etc. Many methods are used to detect and quantify errors in sensor data such as principal component analysis [43,45], artificial neural networks [46,47] and ensemble classifiers [48]. Sensor malfunctions can be identified using three techniques [44]: network-level strategy, homogeneous strategy and heterogeneous strategy.

1. Network-Level Strategy: by using network-level management and tracking the network packets, sensor failures can be detected. This technique is based on Markov models to detect the normal and abnormal sensor response [44].
2. Homogeneous Strategy: this technique uses many identical sensors to detect the malfunction sensor. By arranging the same type of sensors providing the same output, adjacent to each other, the uncorrelated response can be detected, followed by the malfunction sensor [44].
3. Heterogeneous Strategy: this technique merges different types of data points from sensors. By classifying the sensor outputs and training the classifier to find similar data points, the failure is detected using various subsets of sensors [44].

### 2.3. Window Length

This paper addresses the topic of learning from time series data, which can take the form of either raw time sequences or pre-processed tabular data. Examples of pre-processing include the work [49] on linear acceleration, as well as [50,51] on tabular data. Regardless of whether the data are represented as a time sequence or in a feature space, each sample must adhere to a fixed length. The window length should be carefully selected since it affects the classification accuracy, latency and memory size [49,52].

The preferred choice of window size varies in the literature. Generally, the window size varies from 2 s, aiming for real-time decision, to 10 min. The authors in [24] suggested that precise recognition latency increases with the window size. Moreover, methods using Long Short-Term Memory (LSTM) choose a short window length [53], but a too short window brings about inaccurate or unstable recognition. Addressing training with time series data of variable lengths presents significant challenges, as noted in [52], and such methods have yet to be explored in TMD systems to the best of our knowledge. However, we believe that such approaches hold promise for the field, especially for signals exhibiting substantial variations across different time windows. Some initial research was conducted on forecasting transportation modes, as seen in [54], though forecasting itself is considered a separate issue to be examined in a dedicated survey.

### 2.4. Features Extraction for TMD

**Figure 2:**
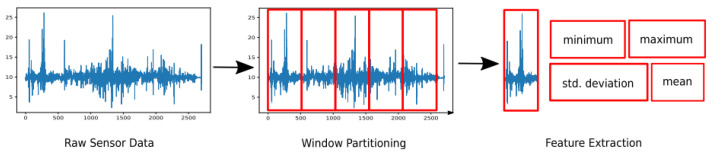
Transforming time series (raw sensor data) into feature space through the segmentation (window partitioning in red) and computation of features (feature extraction (FE)) [35].

Feature computation is the essential component for TMD systems. Each data sample consists of one or more feature components derived from the original time sequence. These features could include metrics such as minimum, maximum, standard deviation (std) and mean, as illustrated in Figure 2. Each individual sample within the dataset is utilized either during the training phase or for evaluation purposes, as discussed in [55].

We synthesize the most used features in the literature that can be computed in each sensor data channel in Table 2.

Several features are often calculated from speed, acceleration and turn angle, such as mean, std, quantile values, quantile ranges and statistics (e.g., kurtosis and skewness). But these features are calculated in a private manner and from different modalities. To summarize, despite the significant development of TMD systems, studies in the literature were carried out relatively independently and each of them established its own transportation mode classification problem and proposed a solution with different parameters, and usually verified it with private datasets which are not publicly available. A fair comparison of results between different groups is very difficult.

### 2.5. Features Optimization

During machine learning models training, the target is to optimize the distribution of samples in the feature space, which represents the relevant variables of the data. This optimization may depend on the training algorithm used, leading to embedded and wrapper methods or to it being independent, resulting in filter-based methods. Several methods are used to optimize this distribution of samples [56,57]. These methods generally aim to minimize intra-class variance, which is the variability of data within the same class, and/or maximize inter-class variance, representing the distance between different classes in the feature space. These two criteria are crucial in developing efficient classifiers. Since these are two important criteria in building classifiers, this section analyzes how the experimental setup and used hardware that is the dataset affect both criteria.

#### 2.5.1. Within-Class Variance in TMD Systems

Within-class variance describes the distribution of samples belonging to the same class. The major risk in TMD studies is to end up with an unrealistically low within-class variance due to highly constrained experimental conditions. Some conditions could be modeled as numerical variables, but others not. Let us start by the numerical variables. Namely, there are the number of involved subjects, their demographic, anthropometric, clinical characteristics (gender, age, height and weight), the type of used devices, the number of different device models, the number of different placements of the sensors on the body, the total duration of the dataset, the duration of the least represented transportation mode, the time span of the data collection campaign and the spatial scale of the data collection. Indeed, they are generally described briefly in the data collection section of TMD studies, but they are rarely quantified. Some of these variables are generally lacking or collected with very low variance in comparison with real data. To illustrate this point, let us consider the HTC dataset discussed in [58], which is regarded as one of the most significant datasets in TMD research to date. This dataset spans a total duration of 8311 h and was collected from 224 subjects, consisting of 110 women and 114 men, who used two different mobile phones. The spatial scale of the collected data is missing. In comparison, ref. [35] collected data from 13 participants, with a total duration of 31 h. A total of 11 different mobile phones were used. On this topic, physiological features are known to be crucial to the field of [59,60], and they are equally important in TMD scenarios that involve physical activity such as walking, biking or riding kick-scooters [61,62].

Certain factors significantly impact within-class variance but are challenging to measure and express numerically. For instance, allowing participants to introduce noises during experiments, simulating real travel conditions, is difficult to quantify. Before training, data cleaning, which involves removing parts of the signal deemed external noise, is almost mandatory. For example, a participant might move their limbs when they are supposed to stay still, or signals could be affected by both vehicle and body movements, leading to ambiguities. Such signals are often removed from the training dataset by experts, a step usually not mentioned in the majority of papers. However, real conditions include these noises, likely decreasing performance in production. Solutions exist in studies on anomaly detection [63,64] but mostly deal with static phases and do not handle combined body and vehicle movements. Therefore, data preparation should be part of the methods, with general solutions suggested for handling unwanted signals from the training stage.

#### 2.5.2. Between-Class Variance in TMD Systems

**Figure 3:**
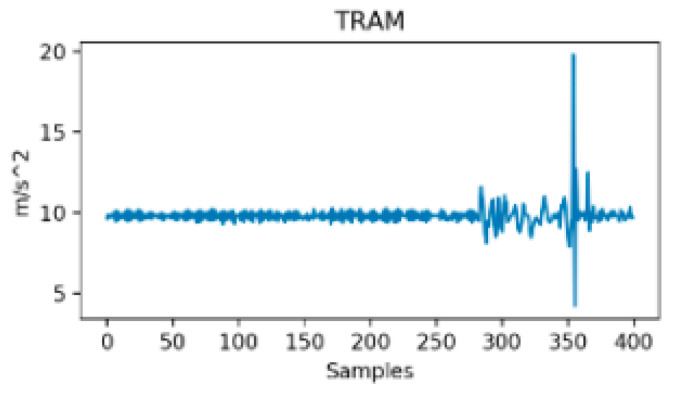
Resultant acceleration in Tram [31].

**Figure 4:**
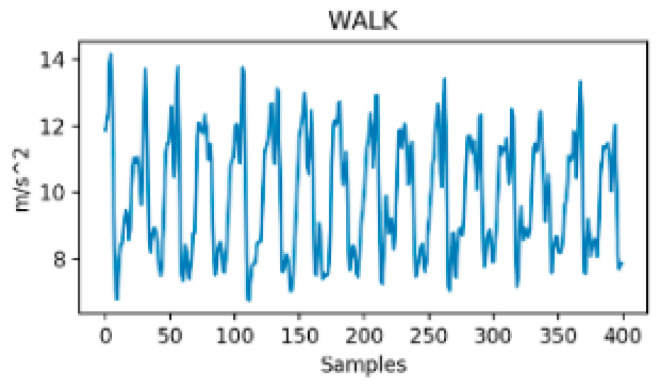
Resultant acceleration in Walk [31].

Between-class variance is commonly an indicator of the distance between means of different classes in the feature space. Unlike within-class variance, between-class variance has a strong dependency on the methodological approach even though both variances may be improved through feature engineering techniques [57]. In the case of TMD systems, between-class variance is mainly determined by the number of considered transportation modes, the nature and number of the used sensors (i.e., signals) and by the feature optimization process if there is one. For instance, two transportation modes could have more or less distinct patterns depending on the considered signals and features. It is expected that, the richer the classification nomenclature, the lower the between-class variance as the probability for blurred borders between classes increases. For instance, a study that considers only vehicle mode versus on-foot activity has a high between-class variance according to vertical acceleration, or to the norm of acceleration. It is, for example, obvious from Figure 3 and Figure 4 that, if the variance (or the std or the range) of both signals is computed through a sliding window of few samples (around 50 corresponding to 1 s in this study), the difference would be important enough between walking and tramway to build an accurate binary classifier.

**Figure 5:**
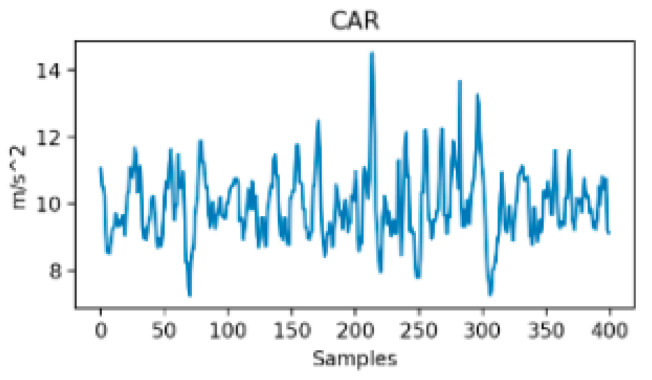
Resultant acceleration in Car [31].

**Figure 6:**
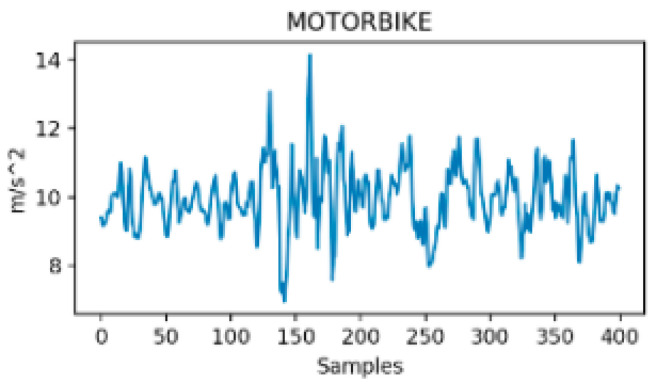
Resultant acceleration in Motorcycle [31].

On the contrary, in Figure 5 and Figure 6, the signals seem similar although they belong to two distinct transportation modes. Moreover, these two figures differ from the previous one, i.e., Figure 3 and Figure 4, in their temporal stability. In fact, for both tramway and walking (0 to 250 s), the signals were almost stationary, meaning they show stable and bounded magnitude variability through time, while being very distinct in terms of magnitude. In the second example, both signals’ magnitudes are close, and the signals show high variability through time that does not seem bounded. As a consequence, building a classifier that distinguishes car from motorcycle is more difficult because the between-class variance is reduced. In this case, the choice of the classifier design is crucial. In addition, such models show higher sensitivity to the train samples due to an increased complexity. However, the main result of this analysis is that the between-class variance, which we recall should be maximized, is much more influenced by the nature of the considered classes rather than by the only number of considered transportation modes.

### 2.6. Categories of Methods for Learning-Based AI

There are three types of machine learning algorithms: supervised, semi-supervised and unsupervised. Supervised learning methods were most frequently employed to detect the transportation mode. These methods require annotated data. Various types of supervised algorithms are used in the literature such as NN, KNN, BN, RF, MLP, DT, SVM and BN algorithms. Semi-supervised learning needs less annotation effort [65,66]. Unsupervised learning, including methods like CNNs and GANs, has shown high accuracy with the absence of labeled data [65,67,68,69,70].

Table 3 makes a summary of the main categories of classification models used for TMD purposes. The methods are separated into eight categories, depending on the two main processes classically undertaken to build a classifier. The first process consists in making feature selection (FS) and the second in choosing either a machine learning (ML) or a deep learning (DL) model-based AI. From the review of the literature, there might be either no feature selection (No FS), a filter-based selection method (FM), a wrapper-based method (WM) or an embedded feature selection method (EMFS). In a few words, FM is undertaken beforehand and independently from the classification model and they generally are based on statistical tests on the similarity between independent features and the output labels. Examples of filter-based methods are Mutual Information (MI) and Maximum Relevance Minimum Redundancy (MRMR). Wrapper-based methods are realized recursively using multiple batches of different feature sets. Given a certain number of features, different combination sets are tested and a classifier is build with each set. The optimal feature set is the one that yields the highest accuracy. In this case, the process can be either entirely automated, such as in Recursive Feature Elimination (RFE), or manual; for example, in [35]. In the latter, different sets of features are fixed and a classifier is built with each set of features. The optimal set of features is that which gives the best classification accuracy. Manual selection is much more efficient in many cases, because it yields satisfying results despite a lower computational cost as compared with RFE, which gradually decreases the number of used features. More globally, wrapper-based methods perform better than filter-based methods because they handle co-dependencies between different input features. Furthermore, unlike FS methods, they are specifically fit to the chosen classification model. As for embedded methods, they are inherently provided by the chosen classifier. For instance, Random Forests (RF) use the split criterion while building the different trees in order to rank features from most to least important.

In Table 3, we separated ML from DL methods, widely used as artificial intelligence techniques in TMD systems. A major observation is the general absence of FS with DL. In fact, neural networks are designed to perform internal feature optimization through the processes of normalization and weighting of the inputs. We view neural networks as a combination of both FE and FS. In fact, the input features are totally transformed into a new set of variables, generally with a lower dimension which is exactly the objective of FE. On the other hand, the weighting of the inputs is, at the same time, a way of ranking features, which is an indirect way of selection. As for ML, an additional layer of feature engineering is commonly added to the classifier. A popular algorithm that has systematically shown promising results is RF, which is an embedded FS method. Second, statistical models such as NB or KNN, as well as geometrical classifiers such as SVM, are combined with the wrapper-based method. The latter generally consists in testing different combination sets of features. The last column titled BM, standing for “Best Model”, provides the model selected in each study after comparing different algorithms. Globally, the two competing algorithms are RF and CNN. Hence, it seems that they must be priority tested while building TMD models.

### 2.7. Performance Evaluation in Classification

Performance evaluation of a classifier is commonly measured through four different metrics:

- Precision: it calculates the proportion of samples properly classified as positive out of all samples classified as positive [1,2].
- Recall: it calculates the proportion of samples correctly classified as positive out of the total actual positives [1,2].
- F1-score: it combines precision and recall in a single value. This metric is used when there is an uneven class distribution and we need to find a balance between precision and recall [1,2].
- Accuracy: it calculates the percentage of correct predictions divided by the total number of predictions [1,2]. It summarizes the overall classification performance for all classes. It is a commonly used metric to assess the performance of a classification model. Determining the suitable machine learning algorithm in a supervised classification considerably affects the accuracy. There are three methods employed to evaluate a classifier’s accuracy. The first method is to divide the training dataset in two-thirds for training and the third for testing. The second method is the cross-validation technique [72], where the training dataset is divided into equal-sized subsets, and for each subset the classifier is trained on the combined data of all the other subsets. The error rate of the classifier is calculated by taking the average of the error rate of each subset. The third method is the leave-one-out validation [73], which is a particular case of the cross-validation method in which all validation subsets contain only one sample. Although this type of validation needs more computational resources, it is important when a precise estimation of a classifier’s error rate is needed [74].
- The influence of training data quantity is important for a classifier’s accuracy [75]. Having a large amount of data provides the machine learning algorithm with more information, enabling the identification of different scenarios and correlation between them before making predictions. As a result, the accuracy will increase.

### 2.8. Overview of Previous Studies on TMD Systems

Table 4 gives a summary of recent studies which address TMD systems. Studies are classified into three families: IMU-based, localization-based and hybrid approaches.

IMU-based approaches used inertial sensors such as accelerometers, gyroscopes, magnetometers, etc., to predict the transportation mode of the user [9,18,24,29,32,33,76,77,78,79]. Localization-based approaches used the GPS receiver to detect the location of the mobile device [39,69,80,81,82,83,84,85,86,87,88]. Hybrid approaches combine inertial and GPS sensors [27,31,89].

We analyze the state of the art from eight aspects: sampling frequency, classified mode, sensors, features, dataset, classifier, window size and accuracy. We included only the accuracy as a measure in Table 4 because it is the most widely used metric to measure the performance of a classifier, which allows for an easy comparison between the methods. Missing data are denoted “-”. These notations were used for convenience: Features, Mag: magnitude, Max: maximum, Min: minimum, Std: standard deviation, var: variance, FFT: Fast Fourier Transform, RMS: Root Mean Square, avg: average. We identified that the authors opt for lower sampling frequencies (between 10 and 50 Hz) for accelerometers, gyroscopes and magnetometers. This reduces battery consumption and the effort of the annotation in the case of supervised learning method.

**Table 4:** Overview of previous studies on travel modes detection.

Approach	Ref	Sampling Frequency [Hz]	Classified Mode	Sensors	Features	Dataset	Classifier	Window Size	Accuracy (%)
IMU	[29]	100	S, W, R, Bi, C, B, T, Sub	A, G, M, B	Mag, jerk, max, min, std, mean, var, kurtosis, skewness, energy, and entropy	SHL2018 [71]	RNN with LSTM	5 s	67.5
	[76]	10	B, Sub, HSR, elevator	A, G, M	Max, mean, range, std, RMS, mean-cross rate, zero-cross rate, slope sign change, spectral centroid, spectral flatness, spectral spread, spectral roll-off, and spectral crest	HTC dataset [58]	LSTM	12 s	97
	[24]	10	B, Sub, HSR, elevator	A, G, M	Mag, max, min, mean, range, std, root mean, cnt zero, cnt mean, cnt slope, spectral centroid, spectral flatness, RMS, max index and max rate	Smartphones’ sensors	LSTM	10 s (elevator), 60 s otherwise	92
	[77]	50	S, W, Bi, B, C, T, Sub	A	Mag and FFT	Accelerometer sensors in smartphones	CNN	10.24 s	94.48
	[18]	20	B, W, C, Bi, T, Tr, Sub, boat, plane	A G	Min, max, avg, and std	Applications	Random forest, random tree, Bayesian network, and naïve Bayes	5 s	95
	[9]	-	S, W, R, Bi, C, B, M, T	A,G,M	Mean, std, highest FFT value	HTC dataset	ANN	17.06 s	87
	[32]	50	S, W, C, T, B	A,G	Min, Max, avg, std	Sensors of smartphones	Bi-LSTM	2.56 s	92.8
	[78]	1	S, W, R, bike, C, B, T, Sub	M, A, G, and pressure sensor	Mean value of the 3 or 4 axes of acceleration, mag, O, gravity and LA, temperature, pressure, altitude	SHL dataset	Bidirectional Encoder Representations from Transformers BERT	-	98.8
	[79]	20	W, S, T, C, B	A, G, LA, O, S, G	Min, max, mean, std	Smartphone embedded sensors	Stacked learning technique (12 machine learning algorithms)	5 s	>89
	[33]	0.067 and 0.2	W, Bi, C, B, T, Sub	A, G	(Max, avg) resultant acceleration, std, skewness, kurtosis, pitch and roll (gyroscope)	Sensor’s smartphone	Random forest	10 min	95.40, 98.78
Localisa- tion	[80]	1	W, Bi, B, C	GNSS	Jerk, mean, std, (10th, 50th, 90th) percentile, skewness	Android app	KNN, RF, MLP	30 s	>74
	[81]	-	W, Bi, C, Bus	GPS	Speed, acceleration, jerk, bearing rate	Geolife data [90]	LSTM	-	83, 81
	[82]	1	W, Bi, Tr, B, taxi, C	GPS	Time, latitude, longitude, altitude, speed	Smartphones sensors	Decision tree	-	94.9
	[83]	Freq max = 1	W, Bi, B, C, MC	GPS, WiFi cellular	Altitude, latitude, longitude, precision, acceleration	37 volunteers in Rio de Janeiro	Hierarchical classifier	60, 90, 120 s	>40
	[84]	-	W, Bi, B, C, T	GPS	Length, mean, covariance, top three velocities and top three acceleration from each segment, speed	GeoLife dataset [90]	Genetic programming	-	>77
	[85]	-	W, Bi, B, T, C	GPS	Speed, altitude, turning angle, net displacement, distance	GPS-enabled mobile applications	Extreme gradient boost, multilayer perceptron	-	96
	[86]	-	W, Bi, B, C, T	GPS	(Avg, min, max) speed, acceleration, jerk, distance, bearing rate, turning change rate, time difference, total duration	GPS tracking data	LSTM	-	93.94
	[87]	-	W, Bi, B, C, T	GPS	Speed, acceleration, jerk, bearing	GPS tracking data	k-mean	30 s	>52
	[39]	-	W, Bi, C, B, T	GPS	(Avg, mean, max) speed, total distance, total time, avg bearing	GPS tracking data	K-means clustering with the ANP-PSO hybrid method	-	88
	[69]	1	W, Bi, B, C, T	GPS	Speed, acceleration, jerk, bearing rate	GeoLife data set [90]	unsupervised deep learning	-	86.7
	[88]	-	W, Bi, C, T, B	GPS	Date, time, longitude, latitude, speed, average speed, average acceleration, maximum and minimum speed, acceleration during each segment, segment distance, direction, duration, bearing	GPS tracking data of 20 different people in Falun	Random forest	300 s	99
Hybrid	[31]	50	MC, W, B, Sub, Tr, S, Car	GPS, A, G	mean, std, skewness, kurtosis, (5th, 95th percentile), avg	sensor readings	CNN, Nearest Neighbor; RF, Statistical Analysis, SVM	2 s	>75
	[27]	GPS (1 Hz), A, G, M (100 Hz)	S, W, Bi, R, B, C, T, Sub	A, G, M, GPS	mag, mean, std, energy, kurtosis, skewness, highest FFT value, frequency	SHL dataset	Decision tree	5.12 s	>50
	[89]	50	S, W, R, Bi, C, B, T, Sub	A, G, M, GPS	-	SHL and TMD dataset [91]	Hybrid DL classifier	-	>90
	[92]	-	Bi, public transport	A, GPS, heart rate data	Mean, median, std, min, max, 10th and 90th percentiles	126 participants living in the Ile-de-France region	Random forest	-	65% for public transport and 95% for biking

## 3. Sensor Types and Locations for TMD Models

**Figure 7:**
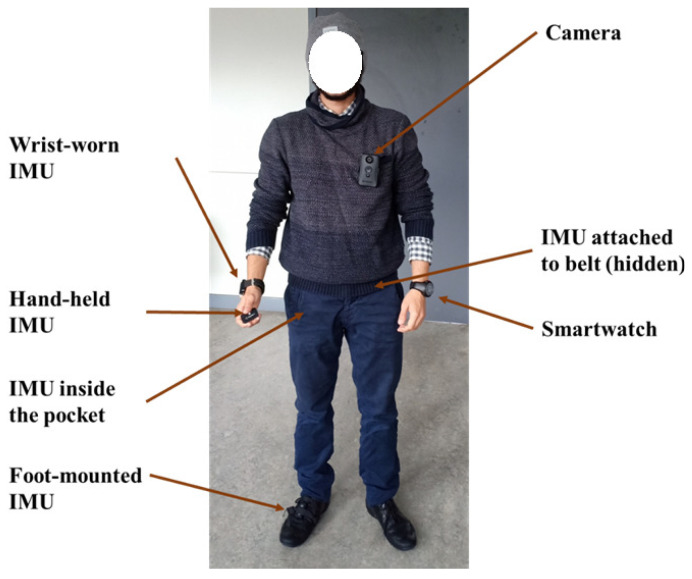
Sensor placement for the perscido dataset [23].

**Figure 8:**
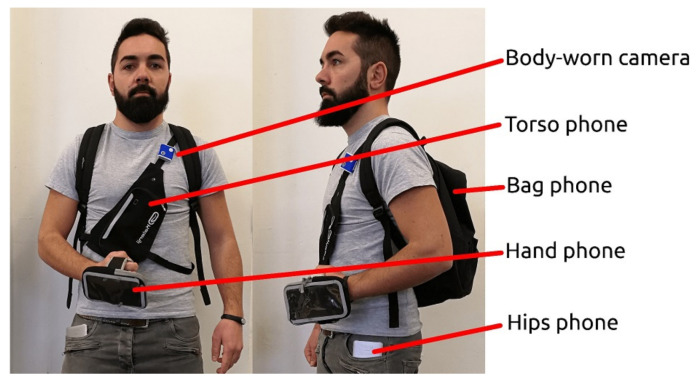
Sensor placement for the SHL dataset [27].

Hardware, for TMD data collection, is key in training TMD models. It is influenced not only by the type of used sensors but also by the chosen device. In fact, two devices may embed the same sensors but exhibit different measurement errors. For example, there are studies that utilize dedicated IMUs for motion analysis [23,93,94,95,96]. The used IMUs in [23] are from the Gaitup Physilog5 series [97], integrating a 3-axis accelerometer, a 3-axis gyroscope and a barometer. These dedicated IMUs generally have more stable frequencies and bounded errors. Additionally, the position of the sensors is crucial, as shown in Figure 7 (on hand, on foot, in pocket, etc.). Recently, smartphones have been equipped with IMUs (and other sensors) enabling data collection (see Figure 8). For instance, in [24], authors use smartphone GPSs to detect common public transportation modes (bus, subway, HSR) and elevator scenarios. In [77], the authors propose using the smartphone accelerometer sensor to detect seven transportation modes. Additionally, authors in [32] use gyroscope and accelerometer sensors for the same purpose. A number of Android applications have been developed specifically to collect data from these sensors.

## 4. Existing Android Applications for TMD Data Collection

**Figure 9:**
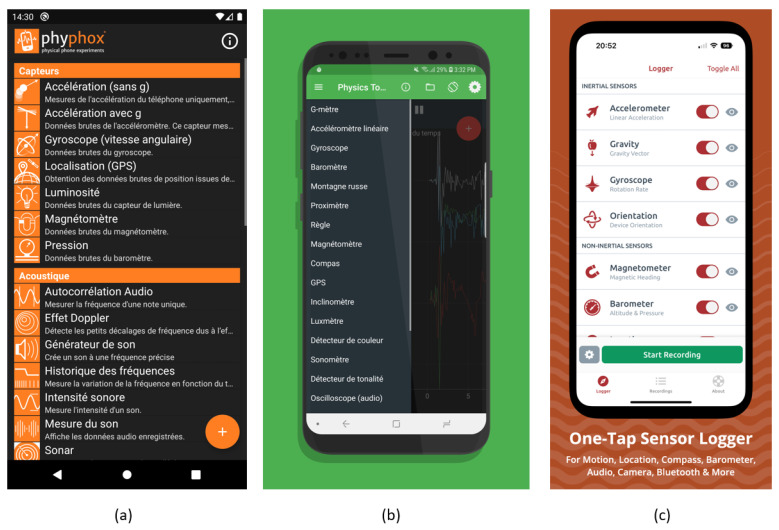
Android applications: (**a**) Phyphox, (**b**) Physics toolbox suite and (**c**) Sensorlogger.

There are mobile applications, displayed in the Google play store, developed to record smartphone sensor data (accelerometer, gyroscope, magnetometer, etc.). These applications are useful for TMD purposes. For example, we cite phyphox, physics toolbox suite and sensorlogger (see Figure 9). They include tools for analyzing and visualizing the collected data directly within the application. Users can record the data for later analysis. Data can be exported in different formats (CSV, Excel, etc.…) for further analysis and sharing. Although these applications are powerful and accessible tools for scientific experimentation, their limitations related to smartphone sensors and data reliability need to be taken into account. Moreover, these applications can collect data, but they are not designed to predict modes of transport. Authors may need to adapt them and add predictive functionality by integrating classification models.

In contrast, several Android applications, more oriented TMD applications, for data collection exist in the literature, but are not present in the Google play store [98,99]. For instance, in [18], the authors suggested a game using online TMD to offer bonuses and impose penalties to users according to their daily transportation mode choice. In [22], the authors developed a mobile application to identify user’s transportation modes based on smartphone sensors. The authors in [98] developed a smartphone system based on person mobility survey to collect data. The system consists of three elements: data collector, data processor and data validator. The data collector is a smartphone application for gathering GPS trajectories, the data processor is a server equipped with rule-based algorithms to analyze travel mobility details and the data validator is a webpage to show the self-collected mobility data for users’ confirmation. The authors in [99] developed a system called edgeTrans. It consists of a smartphone application, a dataset and a server. The dataset records the completed trips. The server executes a machine learning algorithm that created a model which is then added to the edgeTrans system. The installed application identifies the used transport mode offline. In [53], the authors developed a mobile application to identify people’s transportation modes and duration. The implemented mobile application could predict eight classes including stationary, walking, car, bus, tram, train, metro and ferry.

## 5. Standardized TMD Datasets

According to the literature, it is currently challenging to propose a standard algorithm for TMD systems due to the variety of datasets, scenarios and applications. However, the authors in [19,27,100] recommend to use certain datasets as benchmarks in order to determine the optimal algorithm for specific transport mode recognition scenarios. Nevertheless, creating publicly available benchmark datasets, enabling researchers to test and compare their methods, has been difficult until now, since the datasets are collected from different devices and sensors, and even the location of the sensors on the body has an important impact. The authors select and process existing datasets depending on their needs. There is no convincing argumentation for the existence of such an approach, and it is impossible for now, to the best of the authors’ knowledge, to find a common base.

## 6. Conclusions

Enhancing the effectiveness of TMD systems is a current and challenging research area due to many issues. In this review, we aimed to clarify the TMD process, from data collection to classification, by conducting a thorough state-of-the-art analysis of the different steps involved. We provided insights into the problems and the major existing issues and revealed challenges which significantly affect TMD systems performance. Among the challenges that remain to be fully addressed are the placement of the smartphone, the types of used sensors and the influence of environmental conditions. By acknowledging these complexities, this review aims to guide readers’ and beginners’ efforts in developing more effective TMD systems for smart cities.

## Figures and Tables

**Table 1:** Specifications for existing public TMD datasets.

References	Years	Subjects	Sensors	Freq (Hz)	Device Modes	Sensor Positions	Trans-Portation Modes	Total Duration (h)	Spatial Scale	Time Scale	Minimum Duration
[34]	2020	34	A, G, B	32	IMU	Hand, Wrist, Trousers’ pocket, Waist, Foot	S, W, Sr, E, Bi, B, T, KS	48	Grenoble (France)	3 months	1 h
[31]	2020	18	A, G, M, GPS	50	Mob	Pocket, Hand, Car dashboard	S, W, B, C, T, Sub, MC	140	-	-	-
[35]	2018	13	A, G, M, B, S, L	<20	Mob	-	S, W, B, C, T	31	-	-	1.75 h
[27]	2019	3	A, G, M, GPS	100	Mob	Bag, Hips, Torso, Hand	S, W, R, Bi, C, B, T, Sub	703	London (UK)	7 months	21.5 h

**Table 2:** Statistical features.

Sensors	Features
GPS: speed, acceleration, turn angle, trajectory	Mean, std, sinuosity, range, interquartile range, max, quantile k, three maximum values, three minimum values, autocorrelation, kurtosis, skewness, heading change rate, velocity change rate, stop rate, speed, acceleration, turn angle, trajectory
IMU: accelerometer, gyroscope, magnetometer	Mean, std, mean crossing rate, energy, autocorrelation, kurtosis, skewness, min, max, median, range, quantile k, interquartile range, frequency with highest FFT value, ratio between the first and second highest FFT peaks, FFT value
Barometer: pressure	Spectral centroid, spectral spread, number of zero crossings after scaling, main frequency component, power of the main frequency component, spectral energy at 1 Hz, 2 Hz,…, 10 Hz

**Table 3:** FS: Feature selection, CM: Classification modes, BM: Best model, NB: Naive bayes, BN: Bayesian network, DT: Decision tree, DTb: Decision table, SVM: Support vector machine, BDT: Boosted decision tree, FF NN: Feed-forward neural network, RNN: Recurrent neural network, CNN: Convolutional neural network, Bi-LSTM: Bidirectional Long-Short term memory neural network, kNN: k-nearest neighbors, LR: Logistic regression, J48: Decision tree algorithm also known as C4.5, RT: Random Tree, AdaBoost: Adaptive boosting.

	Machine Learning				Deep Learning			
	**Ref**	**FS**	**CM**	**BM**	**Ref**	**FS**	**CM**	**BM**
No FS	[33]	-	NB, SVM, DT, BDT	BDT	[33]	-	FF NN	-
	[31]	-	SVM	-	[31]	-	FF NN, RNN, CNN	CNN
	[49]	-	NB, J48, kNN, SVM	-	[32]	-	Bi-LSTM	-
	-	-	-	-	[61]	-	CNN	-
	-	-	-	-	[49]	-	CNN	CNN
FM	[71]	MI, MRMR	DT	-	-	-	-	-
WM	[35]	manual	SVM, DT	-	[23]	automated	CNN	CNN
	[22]	manual	NB, BN, kNN, LR, J48, DTb, RT	LR	[35]	manual	FF NN	-
EM	[33]	RF	-	-	-	-	-	-
	[31]	-	RF	-	-	-	-	-
	[23]	-	RF	-	-	-	-	-
	[61]	-	RF	RF	-	-	-	-
	[35]	-	RF	RF	-	-	-	-
	[49]	-	AdaBoost, RF	-	-	-	-	-
